# RBFNN-Enabled Adaptive Parameters Identification for Robot Servo System Based on Improved Sliding Mode Observer

**DOI:** 10.1155/2022/8151132

**Published:** 2022-08-22

**Authors:** Ye Li, Dazhi Wang, Mingtian Du, Shuai Zhou, Shuo Cao, Yanming Li

**Affiliations:** ^1^School of Information Science and Engineering, Northeastern University, Shenyang 110819, China; ^2^Department of Mechanical Engineering, The University of Melbourne, Victoria 3010, Australia; ^3^College of Information Engineering, Shenyang Polytechnic College, Shenyang 110045, China; ^4^China North Vehicle Research Institute, Beijing 100072, China

## Abstract

Effective and accurate parameter identification, especially the identification of load torque, is one of the key factors to improve the control performance of the robot servo system. Sliding mode observer (SMO) has always been a common method for identifying load torque due to its advantages of simple implementation, strong robustness, and fast response. However, due to the discontinuity of the SMO switching function, the system will generate high-frequency chattering, which will reduce the accuracy of load torque identification and affect system performance. In this paper, an adaptive parameter identification method based on an improved sliding mode observer is proposed. A continuous deformation mode of saturation function based on boundary variation is proposed as the switching function to alleviate the chattering phenomenon. Meanwhile, the relationship between the sliding mode gain and the feedback gain of proposed SMO is defined so that it can be selected properly to improve the accuracy of identification, and the radial basis function neural network (RBFNN) is used to adaptively tune the boundary layer gain according to the speed change. Moreover, considering that the identification result of the load torque is related to the moment of inertia and the mismatch of the inertia will cause identification errors, the variable period integration method is proposed to identify the inertia and redefine the calculation period of the load torque and inertia. The effectiveness and superiority of the proposed method are verified by simulation experiments. Experimental results demonstrate that the improved SMO combines observer gain coefficient tuning and inertia matching can smoothly and accurately estimate the value of load torque, which is an adaptive identification method worthy of reference for robot servo system.

## 1. Introduction

With the development trend of global industrial intelligence, high-precision servo systems play an important role in industrial robots, medical equipment, military equipment, and other fields. As the core component of the equipment, servo system needs to accurately control motor in full speed range and have good dynamic performance to meet various complex working conditions. However, due to the internal parameters of the servo motor change and the existence of external disturbance, the motor speed will fluctuate and produce chattering phenomenon. For the purpose of suppressing the effect of parameter change and disturbance to alleviate the chattering phenomenon, the load torque of servo motor should be estimated online [1–7].

The operation of servo system depends not only on the control law but also on the load. In the definition of the control law, the dynamic characteristics of the controller at the operating point are mainly concerned, but the influence of load torque is seldom considered. In fact, the load torque can cause significant disturbance to the operation of the servo system, thus changing the operating behavior of the servo system [8–12]. To avoid this situation, the identification of load torque can achieve satisfactory results. Because the load torque sensor is complex and expensive, it is a good solution to estimate the load torque by observer and control strategy [13, 14].

In recent years, many methods to identify load torque have been mentioned in relevant literature. Common load torque identification methods include Kalman filter [15, 16], reduced-order observer [17, 18], and adaptive control strategy based on disturbance observer [19, 20]. In [21], after considering the extended Kalman filter and the unscented Kalman filter, an extended nonlinear Kalman filter is proposed for online coupling and estimation of the load. In [22], a quaternion-based Kalman filter is proposed for industrial robotic applications to estimate and compensate the dynamics of the load and the experimental results demonstrate the feasibility of the approach and its industrial applicability. However, the load identification method based on Kalman filter has the problem of complex observer structure, which makes it difficult to be applied to the system with large inertial load. In [23], in order to reduce the influence of disturbance factors such as load torque change on system performance, a reduced-order load torque observer is proposed, which significantly improves the system's ability to resist load torque disturbance. However, this method is mostly used in heavy load drive and is not suitable for complex systems. Literature [24] combines extended Kalman filter and adaptive linear active disturbance rejection control, which can quickly measure the shock of sudden load and has strong anti-interference ability. In [25], an adaptive control strategy for load torque identification based on observer is proposed, and the observation error, external disturbance, and internal parameter uncertainty are compensated, which effectively enhances the stability of the system. Although the method has good antidisturbance ability, it relies on the mathematical model of the motor and is less robust to changes in the internal parameters of the motor.

After summarizing the problems of the above methods, the sliding mode variable structure control is introduced to identify the load torque, which is simple in implementation, strong in robustness, and fast in response [26–28]. In [29], an improved SMO load torque adaptive identification method is proposed, which is applied to the motor running under variable operating conditions, and the load has been fluctuating. The optimization problem of the cut-off frequency is studied, and the estimation accuracy is high. Reference [30] proposes a nonlinear load torque identification strategy, which combines traditional PI control with high-order fast terminal sliding mode, and uses the identified torque as the feedforward compensation of the PI controller. In [31], a fuzzy sliding mode speed controller that can identify the load is designed. When the internal parameters of the motor or the external load changes, it can effectively suppress the motor chattering and ensure the robust speed control of the system. It is shown from the mentioned literature that the theory of load torque identification method based on SMO is relatively mature and widely used. However, there are still some problems in practical application, such as the identification accuracy which is not high enough and the existence of chattering phenomenon. In particular, the chattering phenomenon of servo motors will cause terminal vibration of industrial robots, which will seriously affect the working performance and efficiency. In this paper, the structure of the conventional SMO is improved and the observer gain coefficient is tuned to solve the chattering problem.

The moment of inertia is needed in the calculation of load torque and it is regarded as a fixed value in most of the studies, while the inertia will change with the dynamic response of the system in practice. The mismatch of inertia will affect the identification of load torque, so it is necessary to carry out online identification of inertia [32]. The commonly used methods of inertia identification include high-order observer [33], multialgorithm compound structure [34], integral method [35], and various optimized neural networks [36]. Compared to other methods, the integral method can overcome the influence of interference and identify inertia smoothly and periodically and has strong antinoise ability; therefore, it is an appropriate method of inertia identification. Literature [37] utilizes the principle that the sinusoidal speed and inertia are out of phase and friction torque is in phase, and the accurate inertia value is obtained by integrating the torque in half period under the control of the low-frequency sinusoidal speed. In order to weaken the influence of the encoder quantization error, literature [38] proposes using an improved variable period integration method to identify the inertia online and use the identified inertia value for the self-tuning of the controller parameters. Literature [39] improves the traditional integral method inertia identification by redefining the update conditions of the sampling period and then optimizes the speed measurement value through the improved neural network, making the whole inertia identification process more accurate. Based on the above discussion, this paper also uses the variable period integration method to identify the inertia and considers the working state of the robot servo controller position adjustment mode, redefines the appropriate sampling period, and alternately identifies the load torque and inertia. Simulation and experimental results verify the effectiveness of the scheme.

Combined with the above literature, the main contribution of this paper is to first propose a variable structure sliding mode observer to identify the load torque. Compared with the conventional SMO, this paper adopts a boundary variation saturation function deformation mode adaptive to the speed change to replace the sign function as the switching signal to alleviate the original chattering phenomenon, and the boundary layer adaptively adjusts with speed. At the same time, the relationship between sliding mode gain and feedback gain of SMO is defined, and RBFNN is used to fit the boundary layer gain function, which solves the configuration problem of the important parameters in the proposed SMO. Another contribution of this paper is that, for the inertia mismatch problem, which is often overlooked in the process of load torque identification, the inertia is identified by the variable period integration method, and the inertia and load torque identification periods are defined. Simulation results demonstrate the effectiveness and superiority of the proposed scheme.

The overall structure of the paper is given as follows.  Section 2 formulates the mathematical mode of servo system permanent magnet synchronous motor (PMSM) and the conventional SMO design method.  Section 3 presents the improved SMO design combining observer gain coefficient tuning and inertia matching.  Section 4 presents the overall design of the robot servo system.  Section 5 presents the simulation experiment part.  Section 6 presents the conclusion.

## 2. Conventional SMO Design

### 2.1. Mathematical Model of Servo System PMSM

PMSM, as the drive device of the servo system, first of all, must be mathematically modeled, and its voltage equation in *d*/*q*-coordinates can be expressed as

(1) $$\begin{array}{c} \left\{\begin{array}{c} u_{d}=Ri_{d}-L_{q}i_{q}\omega_{e}+L_{d}\frac{di_{d}}{dt}\\ u_{q}=Ri_{q}+\left(L_{d}i_{d}+\psi_{f}\right)\cdot \omega_{e}+L_{q}\frac{di_{q}}{dt} \end{array}\right., \end{array}$$

where *u*_*d*_ and *u*_*q*_ are the *d*/*q*-axis voltages, *R* is the stator resistance, *L*_*d*_ and *L*_*q*_ are the *d*/*q*-axis inductances, *ω*_*e*_ is the electrical angular speed of motor, *i*_*d*_ and *i*_*q*_ are the *d*/*q*-axis currents, and *ψ*_*f*_ is the rotor permanent magnet flux-linkage.

The electromagnetic torque equation of PMSM can be expressed as

(2) $$\begin{array}{c} T_{e}=\frac{3}{2}P_{n}\cdot \left[\psi_{f}+\left(L_{d}-L_{q}\right)i_{d}\right]\cdot i_{q}, \end{array}$$

where *T*_*e*_ is the electromagnetic torque and *P*_*n*_ represents the pole pairs of motor.

The mechanical motion equation of PMSM can be expressed as

(3) $$\begin{array}{c} J\frac{d\omega_{m}}{dt}=T_{e}-B\omega_{m}-T_{L}, \end{array}$$

where *J* is the moment of inertia, *B* is the viscous friction coefficient, *T*_*L*_ is the load torque, and *ω*_*m*_ is the mechanical angular speed of the motor.

### 2.2. Design of the Conventional SMO

Substituting equations (2) and (3), the state equation of PMSM can be expressed as

(4) $$\begin{array}{c} d\omega_{m}/dt=\frac{3}{2}P_{n}\cdot \left[\psi_{f}+\left(L_{d}-L_{q}\right)i_{d}\right]\cdot i_{q}/J-B\omega_{m}/J-T_{L}/J. \end{array}$$

Based on this, the conventional SMO with speed as observation object can be described as

(5) $$\begin{array}{c} d{\hat{\omega }}_{m}/dt=\frac{3}{2}P_{n}\cdot \left[\psi_{f}+\left(L_{d}-L_{q}\right)i_{d}\right]\cdot i_{q}/J-B{\hat{\omega }}_{m}/J-G_{s}, \end{array}$$

where ${\hat{\omega }}_{m}$ is the estimated mechanical angular speed, ${\hat{T}}_{L}$ is the estimated load torque, $G_{s}=k\cdot \mathrm{sgn}\left({\hat{\omega }}_{m}-\omega_{m}\right)$ is the switch signal function defined by sign function sgn(*x*), and *k* is the sliding mode gain.

The speed estimation error is defined as ${\tilde{\omega }}_{m}={\hat{\omega }}_{m}-\omega_{m}$, and, by subtracting (4) from (5), the following can be obtained:

(6) $$\begin{array}{c} d{\tilde{\omega }}_{m}/dt=T_{L}/J-B{\tilde{\omega }}_{m}/J-G_{s}. \end{array}$$

Define the sliding surface as

(7) $$\begin{array}{c} S={\tilde{\omega }}_{m}={\hat{\omega }}_{m}-\omega_{m}. \end{array}$$

According to the SMO control theory, when the system enters the sliding mode, it satisfies $S=\dot{S}=0$ and the load torque on the basis of (6) can be estimated as

(8) $$\begin{array}{c} {\hat{T}}_{L}=G_{s}\cdot J. \end{array}$$

The sliding mode control function becomes discontinuous due to the introduction of sign function, which results in high frequency noise in estimated load torque. Therefore, the actual estimated load torque can be expressed as

(9) $$\begin{array}{c} {\hat{T}}_{L}=T_{L}+\Delta n_{s}, \end{array}$$

where Δ*n*_*s*_ is the high frequency noise. The existence of high frequency noise will cause the chattering phenomenon to the SMO and degrade the identification performance of the load torque.

**Figure 1:**
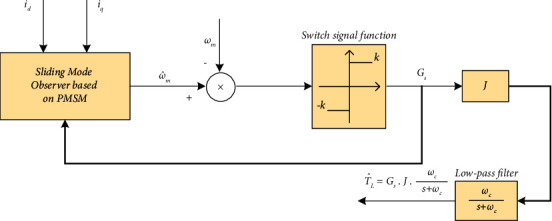
The principle diagram of conventional SMO to identify the estimated load torque.

For suppressing the chattering phenomenon of the load torque identification process, a low-pass filter (LPF) is introduced to suppress the chattering signal. After all, the estimated load torque can be expressed as

(10) $$\begin{array}{c} {\hat{T}}_{L}=G_{s}\cdot J\cdot \frac{\omega_{c}}{s+\omega_{c}}, \end{array}$$

where *ω*_*c*_ is the cut-off frequency of the LPF. The principle diagram of conventional SMO to identify the estimated load torque is shown in Figure 1.

However, the introduction of the LPF will cause problems of system delay and identification error, which degrades the identification performance. Meanwhile, based on the above analysis and derivation, the estimated load torque is related to the moment of inertia. If the inertia changes so that the parameters mismatch, it also affects the identification performance of the system. Therefore, for the purpose of solving the above problems, a switch function with better continuity to replace the sign function and real-time identification of moment of inertia are needed.

## 3. Improved SMO Combines Observer Gain Coefficient Tuning and Inertia Matching for Load Torque Identification

According to the above analyses, the conventional method of load torque identification based on SMO produces high frequency noise and causes the chattering phenomenon that reduces the performance of the system. In the meantime, another reason for the degradation of system identification performance is the mismatching of moment of inertia. To solve these problems, this paper proposes an improved SMO identification method by introducing a continuous deformation mode of saturation function based on boundary variation as the switching function combines observer gain coefficient tuning and inertia matching. Detailed description of the proposed method is presented in this section.

### 3.1. Design of the Improved SMO

The main reason for the chattering phenomenon is the system discontinuous jump caused by the sliding mode control law during the switching action; especially when the switching function is sign function, it is easy to cause the system to switch back and forth discontinuously near *S*=0. Therefore, the output of SMO needs to filter high frequency noise through LPF, but this will cause time delay to the system. To eliminate this unexpected chattering and serialize discontinuous switch items as much as possible, a boundary mutation of the saturation function deformation mode with boundary layer as independent variable is proposed as the switching function in this paper. Compared with the sign function, the continuity of saturation function is better and the output of SMO is smoother which can effectively alleviate the chattering phenomenon. The proposed saturation function *Sat*(*S*) can be expressed as

(11) $$\begin{array}{c} Sat\left(S\right)=\left\{\begin{array}{cc} \mathrm{sgn}\left(S\right) & \left|S\right|\geq \phi \\ \tanh\left(2\pi S/\phi \right) & \left|S\right|<\phi \end{array}\right., \end{array}$$

where $S={\hat{\omega }}_{m}-\omega_{m}$, *ϕ* is the boundary layer, and the switching function *G*_*s*_ can be redefined as

(12) $$\begin{array}{c} G_{s}=k\cdot Sat\left(S\right). \end{array}$$

**Figure 2:**
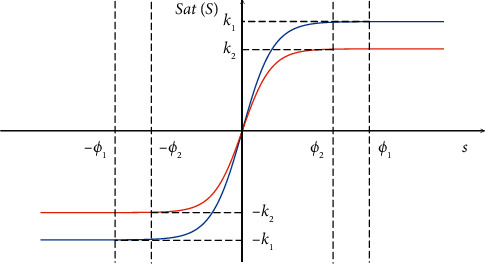
The diagram of the saturation function.

If the boundary layer is unchanged, with the increase of the response time, the system will generate a lot of chattering at high speed. Therefore, in order to guarantee the switching response time, the boundary layer width needs to become wider as the speed increases. The diagram of the saturation function is shown in Figure 2, where *ω*_*ϕ*_1__ > *ω*_*ϕ*_2__.

Inspired by [40], an additional component *G*_*a*_ is introduced to represent the average estimated load torque, and the load torque observer can be reexpressed as

(13) $$\begin{array}{c} d{\hat{\omega }}_{m}/dt=\frac{3}{2}P_{n}\cdot \left[\psi_{f}+\left(L_{d}-L_{q}\right)i_{d}\right]\cdot i_{q}/J-B{\hat{\omega }}_{m}/J-G_{s}-l\cdot G_{a}, \end{array}$$

where *l* is the feedback gain and *G*_*a*_ can be obtained through an LPF and expressed as

(14) $$\begin{array}{c} G_{a}=G_{s}\cdot \frac{\omega_{c}}{s+\omega_{c}}. \end{array}$$

After introducing the average estimated load torque, according to (13), the speed estimation error can be expressed as

(15) $$\begin{array}{c} d{\tilde{\omega }}_{m}/dt=T_{L}/J-B{\tilde{\omega }}_{m}/J-G_{s}-l\cdot G_{a}. \end{array}$$

According to SMO control theory, when the system enters the sliding mode that satisfies $S=\dot{S}=0$, the estimated load torque can be expressed as

(16) $$\begin{array}{c} {\hat{T}}_{L}=\left(G_{s}+l\cdot G_{a}\right)\cdot J. \end{array}$$

It follows that (16) is divided into two parts: *l* · *G*_*a*_ · *J* is the low-frequency effective component of the estimated load torque filtered by LPF, while *G*_*s*_ · *J* is the harmonic component generated by switching function. Since the LPF filters the estimated effective component of the load torque, its response delay will affect the accuracy of the system identification under dynamic conditions. Therefore, the error of load torque estimation will be compensated in the following research.

### 3.2. Observer Gain Coefficient Tuning

Based on above analysis, the appropriate selection of the sliding mode gain, feedback gain, and boundary layer value is significant for the accuracy of load torque identification. Larger or smaller parameters selection can cause chattering phenomenon or reduce dynamic performance of the system. Therefore, these parameters are selected appropriately in this paper.

According to the sliding mode control theory, the sliding mode reachability condition is $S\cdot \dot{S}<0$. It should be noted that when the error value of the speed estimation oscillates around the zero point of the sliding mode surface, the values of the switching functions are different. Therefore, combine equations (14)–(16) and consider the situation of *S* ≥ *ϕ*, −*ϕ* < *S* < *ϕ*, and *S* ≤ −*ϕ*; the reachable condition of the sliding mode can be expressed as

(17) $$\begin{array}{c} S\cdot \dot{S}=\left\{\begin{array}{cc} S\cdot \left[\frac{T_{L}-B\cdot S}{J}-\left(1+l\cdot \frac{\omega_{c}}{s+\omega_{c}}\right)\cdot k\right] & S\geq \phi \\ S\cdot \left[\frac{T_{L}-B\cdot S}{J}-\left(1+l\cdot \frac{\omega_{c}}{s+\omega_{c}}\right)\cdot k\cdot \tanh\left(2\pi S/\phi \right)\right] & -\phi <S<\phi \\ S\cdot \left[\frac{T_{L}-B\cdot S}{J}+\left(1+l\cdot \frac{\omega_{c}}{s+\omega_{c}}\right)\cdot k\right] & S\leq -\phi \end{array}\right\}<0. \end{array}$$

Because inertia *J* and the viscous friction coefficient *B* are all positive numbers, −*B* · *S*/*J* < 0. Similarly, because tanh(2*πS*/*ϕ*) · *S* > 0, −(1+*l* · *ω*_*c*_/*s*+*ω*_*c*_) · *k* · tanh(2*πS*/*ϕ*) · *S* < 0. In conclusion, the sufficiently unnecessary condition satisfying (17) can be expressed as

(18) $$\begin{array}{c} \left(1+l\cdot \frac{\omega_{c}}{s+\omega_{c}}\right)\cdot k>T_{L}/J. \end{array}$$

On the basis of (18), the selection condition of feedback gain can be expressed as

(19) $$\begin{array}{c} l>\left(\frac{T_{L}}{J\cdot k}-1\right)\cdot \frac{s+\omega_{c}}{\omega_{c}}. \end{array}$$

In order to simplify the calculation process while satisfying the realization of sliding mode motion, remove the second term to the right of (19), and the relation between the sliding mode gain and the feedback gain can be expressed as

(20) $$\begin{array}{c} l=2T_{L\max}/\left(J\cdot k\right)-1, \end{array}$$

where *T*_*L*max_ is the motor maximum load torque. It can be seen from (20) that when the value of sliding mode gain or feedback gain is given, the value of the other parameter can be obtained.

As mentioned above, when the speed changes dynamically, the chattering occurs near the sliding mode surface if the boundary layer of the saturation function is fixed. A lot of chattering will occur as response time increases at high speed and the steady-state error increases at low speed. Thus, the boundary layer of a saturation function which can be adjusted dynamically with speed changes can alleviate the chattering phenomenon when the system runs at different speed. The relationship between boundary layer and speed can be expressed as

(21) $$\begin{array}{c} \phi =m\cdot \left({\hat{\omega }}_{m}-\omega_{m}\right), \end{array}$$

where *m* is the boundary layer gain.

To obtain an accurate relationship between boundary layer gain and speed dynamics so that the observer chattering is the weakest, RBFNN is adopted to approximate the function of (21) in which the boundary layer gain can be adaptively estimated with the speed dynamics in this paper. As a powerful tool for defining uncertain systems, adaptive neural networks are widely used to solve the problems of unknown systems and functions [41–45]. Similarly, RBFNN is a feedforward neural network with the unique best approximation, simple training, and fast learning convergence. It can approximate any continuous nonlinear function with arbitrary precision. The steps of the RBFNN algorithm are as follows:

Step 1: define *ω*_*m*_ and ${\hat{\omega }}_{m}$ as the input of the RBFNN and $x={\left[\omega_{m},{\hat{\omega }}_{m}\right]}^{Τ}$.
Step 2: select Gaussian function as hidden layer function and it can be expressed as
  (22) $$\begin{array}{c} \varphi_{i}\left(x\right)=\exp\left(-\frac{{\left\|x-\mu_{i}\right\|}^{2}}{\sigma_{i}^{2}}\right)i=1,2,\cdots M, \end{array}$$
where *μ*_*i*_ and *σ*_*i*_ are the center point of the Gaussian function and variance of the *i*th node, respectively. *M* is the number of radial basis neurons in the hidden layer.
Step 3: the boundary layer gain *m* is the output of the RBFNN and the output is calculated as
  (23) $$\begin{array}{c} m_{j}=\sum_{i=1}^{M}w_{ij}\varphi_{i}\;j=1,2,\cdots ,P, \end{array}$$
where *w*_*ij*_ is the weight and *P* is the number of linear neurons in the output layer.
Step 4: train RBFNN with supervised learning algorithm, and define the objective function as
  (24) $$\begin{array}{c} E=\frac{1}{2}\sum_{j=1}^{P}e_{j}^{2}\\ e_{j}=m_{j}-\sum_{i=1}^{M}w_{ij}\cdot \exp\left(-\frac{{\left\|x-\mu_{i}\right\|}^{2}}{2\sigma_{i}^{2}}\right). \end{array}$$
In order to minimize the objective function, the correction amount of each parameter should be proportional to its negative gradient.
Step 5: set the error threshold; if the chattering decreases and the estimation error is less than the threshold, output the boundary layer gain *m*; otherwise, go back to step 1.

By adjusting the appropriate value of *m*, the chattering phenomenon can be alleviated.

### 3.3. Identification of Moment of Inertia

It can be obtained that the value of the estimated load torque is related to the moment of inertia. However, in previous studies, most papers regarded the moment of inertia as a fixed value. In practice, the moment of inertia will deviate with the change of the system running state, especially for industrial robot servo systems that the moment of inertia will change with the attitude of the manipulator. Therefore, in order to avoid the load torque identification error due to the mismatch of inertia, an improved integral method is adopted to identify the inertia in this paper.

First assume that the viscous friction coefficient remains constant. The mechanical motion equation can be rewritten as

(25) $$\begin{array}{c} T_{e}\left(t\right)=\hat{J}\frac{d\omega_{m}\left(t\right)}{dt}+\Delta J\frac{d\omega_{m}\left(t\right)}{dt}+B\omega_{m}\left(t\right)+T_{L}, \end{array}$$

where $\hat{J}$ is the observe value and Δ*J* is the error value caused by the disturbance torque. Multiplying both sides of the equation by the derivative of the speed and integrating equation (25), we have

(26) $$\begin{array}{c} \int_{t_{i}}^{t_{f}}T_{e}\left(t\right)\frac{d\omega_{m}\left(t\right)}{dt}dt=\hat{J}\cdot \int_{t_{i}}^{t_{f}}{\left(\frac{d\omega_{m}\left(t\right)}{dt}\right)}^{2}dt+\Delta J\cdot \int_{t_{i}}^{t_{f}}{\left(\frac{d\omega_{m}\left(t\right)}{dt}\right)}^{2}+B\cdot \int_{t_{i}}^{t_{f}}\omega_{m}\left(t\right)\frac{d\omega_{m}\left(t\right)}{dt}dt+T_{L}\cdot \int_{t_{i}}^{t_{f}}\frac{d\omega_{m}\left(t\right)}{dt}dt, \end{array}$$

where *t*_*i*_ and *t*_*f*_ are the initial and finish times of the integration period, respectively. Arranging (26), the sum of $\hat{J}$ and Δ*J* can be expressed as

(27) $$\begin{array}{c} \hat{J}+\Delta J=\frac{\int_{t_{i}}^{t_{f}}T_{e}\left(t\right)d\omega_{m}\left(t\right)/dtdt}{\int_{t_{i}}^{t_{f}}{\left(d\omega_{m}\left(t\right)/dt\right)}^{2}dt}-\frac{1}{2}B\cdot \frac{\left[\omega_{m}{\left(t_{f}\right)}^{2}-\omega_{m}{\left(t_{i}\right)}^{2}\right]}{\int_{t_{i}}^{t_{f}}{\left(d\omega_{m}\left(t\right)/dt\right)}^{2}dt}-T_{L}\cdot \frac{\left[\omega_{m}\left(t_{f}\right)-\omega_{m}\left(t_{i}\right)\right]}{\int_{t_{i}}^{t_{f}}{\left(d\omega_{m}\left(t\right)/dt\right)}^{2}dt}. \end{array}$$

The numerators of the second and third terms of (27) can be regarded as a constant and if the integration period is long enough, the second and third terms can be approximately equal to zero. Similarly, if the speeds at the initial and finish times of the integration period are equal, the second and third terms can also be equal to zero. However, the long integration period is not suitable for real-time inertia identification and periodic speed condition is too rigorous for industrial robot servo systems because the commands are irregular under normal circumstances, which limits the use of traditional integral methods.

When the robot servo system works in the position regulation mode, it will frequently start and stop reciprocating motion. Therefore, the sampling period is improved by increasing sampling period to make it more suitable for position regulation mode, from which speed is equal at the initial and finish times of the integration period to speed of zero in this paper. The improved sampling period update condition can be expressed as

(28) $$\begin{array}{c} \omega_{m}\left(k\right)=\omega_{m}\left(k-i\right)=\text{0,}\frac{d\omega_{m}}{dt}\neq 0, \end{array}$$

where *k* is the sampling point and *k* ∈ *N*^+^.

Finally, the identification inertia can be expressed as follows:

(29) $$\begin{array}{c} \hat{J}\left(k\right)=\frac{\int_{\left(k-i\right)T_{pi}}^{kT_{pi}}T_{e}\left(t\right)d\omega_{m}\left(t\right)/dtdt}{\int_{\left(k-i\right)T_{pi}}^{kT_{pi}}{\left(d\omega_{m}\left(t\right)/dt\right)}^{2}dt}, \end{array}$$

where *T*_*pi*_ is the inertia identification period. To prove the convergence of (29), the total error value Δ*J*_total_ can be expressed as

(30) $$\begin{array}{c} \underset{k⟶\infty }{\lim}\Delta J_{total}=\Delta J\left(k\right). \end{array}$$

Then,

(31) $$\begin{array}{c} J=\underset{k⟶\infty }{\lim}\hat{J}\left(k\right). \end{array}$$

From (31), it can be seen that using the integral method to identify the inertia will eventually converge to a constant, which proves the convergence of the method.

**Figure 3:**
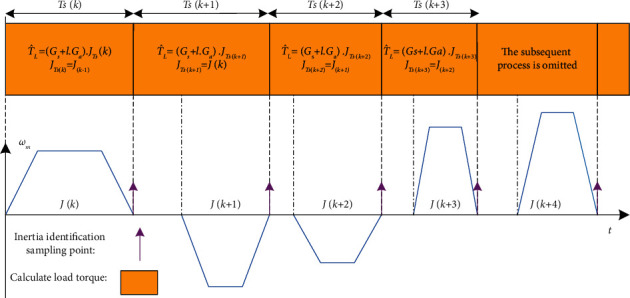
The redefined sampling period timing sequence diagram.

According to the above analysis, the proposed SMO has a prerequisite in the identification process of the load torque, in which the inertia needs to remain unchanged throughout the identification period. Therefore, this paper redefines the sampling period of two-parameter identification. First set the initial value of inertia, and calculate the load torque through the initial value of inertia until the inertia satisfies its update condition. The update condition for inertia is that the speed at the initial and finish time of the integration period is equal to 0, as shown in equation (29). The updated inertia value obtained is used for the estimation of the load torque in the next identification period, until the inertia satisfies the next update condition and the new inertia value is recalculated, and complete a sampling period and repeat the above process. The redefined sampling period timing sequence diagram is shown in Figure 3 and the identification of load torque is always in progress. *T*_*s*_ represents a complete sampling period.

In summary, compared with the conventional method with a fixed sampling period and no consideration of parameter changes, the proposed method redefines the sampling period in this paper, sampling, and identification of inertia and load torque alternately to make it more suitable for position regulation mode of robot servo system. By updating load torque and inertia continuously, the system has more accurate dynamic identification results.

### 3.4. Stability Analysis of the Improved SMO

The stability analysis of the improved SMO proposed in this paper is based on (15). Consider the position of *S* on the sliding surface, respectively, and suppose that the LPF has a high-frequency cut-off that *S* is not affected. When *S* ≥ *ϕ* , the value of the switching signal function is *k*. Similarly, when *S* ≤ −*ϕ*, the value of the switching signal function is −*k*. When −*ϕ* < *S* < *ϕ*, where the error of the speed observation slides near the zero of the sliding mode, the value of the switching signal function is *k* · tanh(2*πS*/*ϕ*). Equation (15) can be expressed as

(32) $$\begin{array}{c} dS/dt-T_{L}/J+B\cdot S/J=\left\{\begin{array}{cc} \;-\left(1+l\right)\cdot k & \;S\geq \phi \\ \;-\left(1+l\right)\cdot k\cdot \tanh\left(2\pi S/\phi \right) & \;-\phi <S<\phi \\ \left(1+l\right)\cdot k & \;S\leq -\phi \end{array}\right.. \end{array}$$

Since *J* and *B* are always positive, when *S* ≥ *ϕ* or *S* ≤ −*ϕ*, the system is always stable according to the Routh-Hurwitz stability criterion. When −*ϕ* < *S* < *ϕ*, all the coefficients in *B* · *S*/*J*+(1+*l* · *ω*_*c*_/*s*+*ω*_*c*_) · *k* · tanh(2*πS*/*ϕ*) are positive, so the system is also stable. Meanwhile, the larger the value of the coefficient, the shorter the transient time of the system. Therefore, according to above analysis, the switching signal function adopted in this paper can not only alleviate the chattering phenomenon of the system but also improve its convergence rate.

## 4. Design of the Servo Drive System

**Figure 4:**
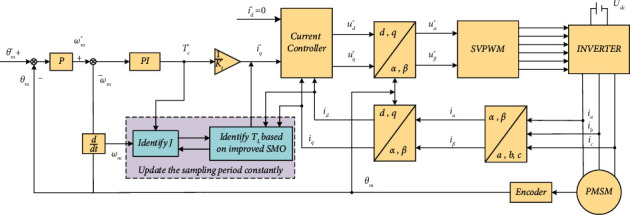
The schematic diagram of the servo drive system.

The design of the servo drive system designed includes the following modules: the position control loop based on *P* regulator, the speed control loop based on *PI* regulator, and the current control loop based on *P* regulator; Park inverse transformation; space vector pulse width modulation (SVPWM); inverter; PMSM; encoder; Park and Clark transformation; and inertia and load torque identification module. The calculated load torque is used to compensate the input current of the current controller. The schematic diagram of the servo drive system is shown in Figure 4.

## 5. Simulation Experimental Verification

In this paper, an SMO with boundary mutation saturation function deformation form which is combined with the moment of inertia matching is proposed to identify the load torque. In order to verify the effectiveness and superiority of the improved method, a simulation experimental model of servo motor drive system based on SMO is designed. The detailed parameters of the servo controller model are descripted in Table 1.

### 5.1. Estimated Performance of the Improved SMO

To validate the effectiveness of the improved SMO, the estimated performance of conventional SMO and improved SMO which adopt the saturation function deformation mode are compared firstly. The speed of the simulation experiment is chosen to be a constant speed of 1000 r/min. The load torque of the simulation experiment is chosen to be a constant load torque of 5 N·m and 10 N·m, respectively. The parameters of the SMO are as follows: *k*=1000, *l*=9, and *ϕ*=20.

**Figure 5:**
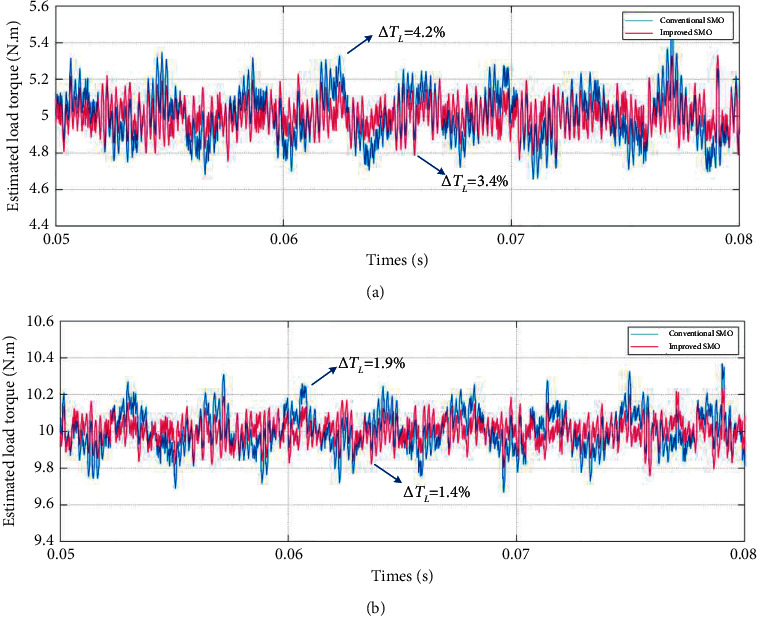
Partial experimental results of load torque estimation performance under the condition of 1000 r/min and fixed boundary layer. (a) Load torque is 5 N·m. (b) Load torque is 10 N·m.

**Figure 6:**
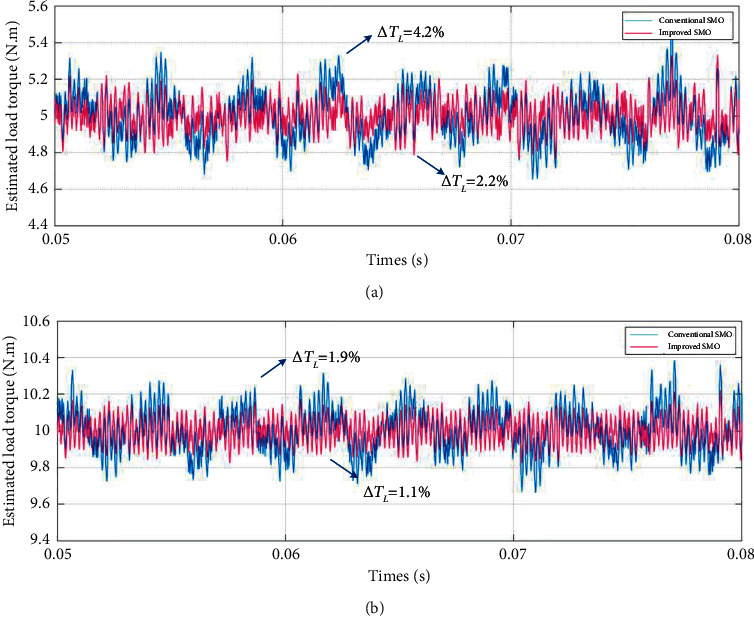
Partial experimental results of load torque estimation performance under the condition of 1000 r/min and boundary layer gain tuning combined with RBFNN. (a) Load torque is 5 N·m. (b) Load torque is 10 N·m.

**Figure 7:**
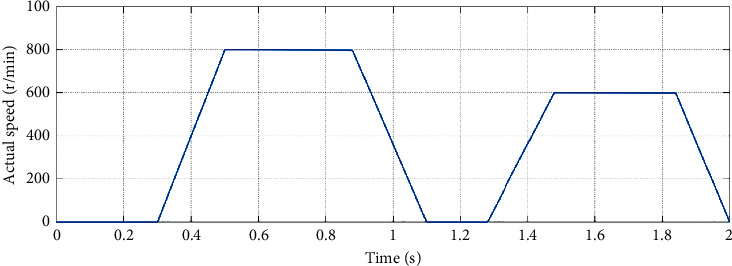
Partial speed waveform that simulates servo system position regulation mode start-stop motion.

**Figure 8:**
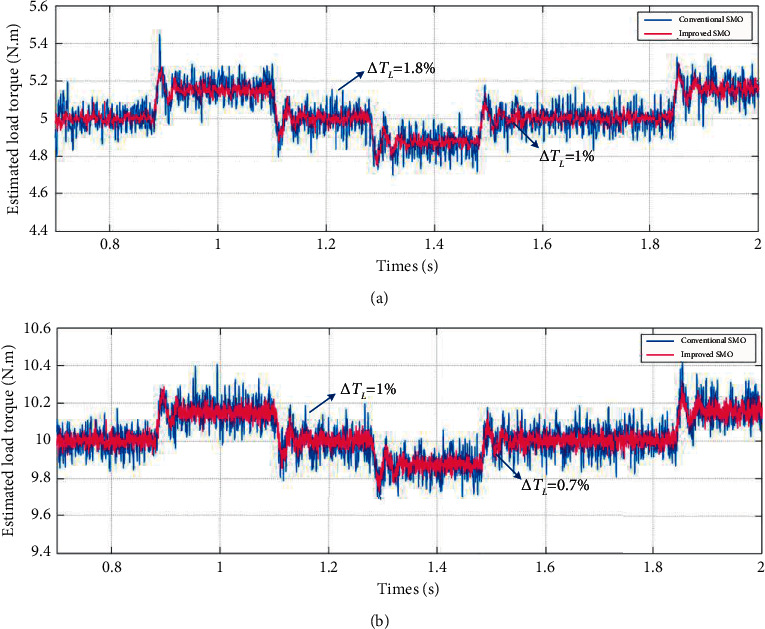
Partial experimental results of load torque estimation performance under the condition of variable speed and boundary layer gain tuning combined with RBFNN. (a) Load torque is 5 N·m. (b) Load torque is 10 N·m.

In this part, the improved SMO in three cases of constant speed with fixed boundary layer, constant speed and variable boundary layer gain tuning combined with RBFNN, and variable speed and variable boundary layer gain tuning combined with RBFNN is compared with the conventional SMO in the estimation performance of load torque by simulation experiments. The simulation results are shown in Figures 5–8. Due to the large number of sampling points, in order to clearly observe the experimental results of load torque identification, all experimental results in this paper are partially enlarged experimental results.

Figure 5 shows the conventional and improved SMO load torque estimation results after the motor runs smoothly. In Figure 5(a), it is calculated from experimental data that the mean error of estimation for conventional SMO and improved SMO is about 4.2% and 3.4%, respectively, when the load torque is 5N·m. In Figure 5(b), it is calculated from experimental data that the mean error of estimation for conventional SMO and improved SMO is about 1.9% and 1.4%, respectively, when the load torque is 10 N·m.

Therefore, it can be proved from the experimental results that when the boundary layer is fixed, the waveform ripples of the improved SMO for load torque estimation are much smaller than those of the conventional SMO under constant speed. The estimated load torque is closer to actual load torque and the chattering phenomenon has eased.

Similarly, the estimated performances of conventional SMO and improved SMO which adopt the saturation function deformation mode with boundary mutation are verified next. The speed is also chosen to be a constant at 1000 r/min. The load torque of the simulation experiment is chosen to be a constant load torque of 5 N·m and 10 N·m, respectively. The parameters of the SMO are as follows: *k*=1000 and *l*=9. Tuning boundary layer gains via RBFNN and the boundary layer gain *m*=10.

Figure 6 shows the conventional and improved SMO load torque estimation results after the motor runs smoothly when the speed is constant. In Figure 6(a), it is calculated from experimental data that the mean error of estimation for conventional SMO and improved SMO is about 4.2% and 2.2%, respectively, when the load torque is 5 N·m. In Figure 6(b), it is calculated from experimental data that the mean error of estimation for conventional SMO and improved SMO is about 1.9% and 1.1%, respectively, when the load torque is 10 N·m.

A speed waveform that simulates servo system position regulation mode start-stop motion in which part of the waveform is shown in Figure 7 is given. The load torque of the simulation experiment and the parameters of the SMO are the same as those set above.

Figure 8 shows the conventional and improved SMO load torque estimation results under the given speed waveform. After the system runs smoothly, it is calculated from experimental data that the mean error of estimation for conventional SMO and improved SMO is about 1.8% and 1%, respectively, when the load torque is 5 N·m as shown in Figure 7(a) and about 1% and 0.7%, respectively, when the load torque is 10 N·m as shown in Figure 7(b).

It is shown from the experimental results that whether the motor is running in constant speed or variable speed, the waveform ripples of the improved SMO for load torque estimation are much smaller than those of the conventional SMO under variable boundary layer gain tuning combined with RBFNN, and the load torque varies with the actual speed of the motor in the process of position regulation mode start-stop motion. The chattering phenomenon of the improved SMO is weaker than that of conventional SMO.

### 5.2. Estimated Performance of the Improved SMO Combined with moment of Inertia Matching

According to the above analysis, the calculation of load torque requires the value of moment of inertia. The uncertainty of the inertia will change the dynamic characteristics of the observer. Therefore, the mismatching of moment of inertia will cause errors in the estimation of load torque.

**Figure 9:**
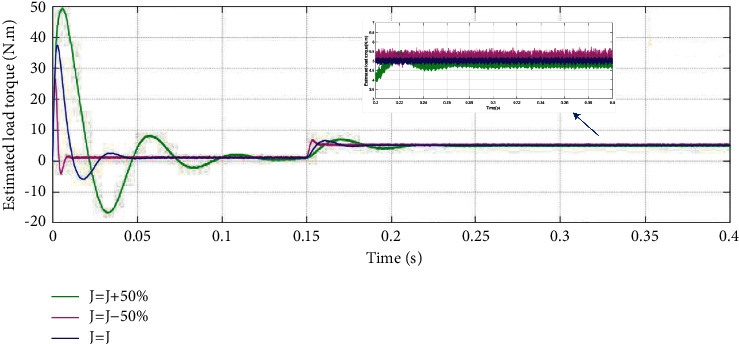
Influence of moment of inertia variation on load torque estimation performance.

First, verify the influence of inertia offset on load torque identification and Figure 9 shows the simulation results. The speed of the simulation experiment is chosen to be a constant speed of 1000 r/min. The initial load torque is 1 N·m and it increases to 5 N·m when the system runs to 0.15 seconds. The parameters of the SMO are as follows: *k*=1000, *l*=9, and *ϕ*=10.

It is shown from the experimental result that when the inertia increases by 50%, the dynamic characteristics of the observer also change, the estimated value of load torque is in error and oscillates, and the steady-state response time becomes longer. When the inertia reduces by 50%, although the steady-state response time is shorter, the observer becomes less sensitive to parameter changes and the estimated value of load torque is also in error and oscillates. Therefore, accurate moment of inertia matching is significant to estimate the load torque.

**Figure 10:**
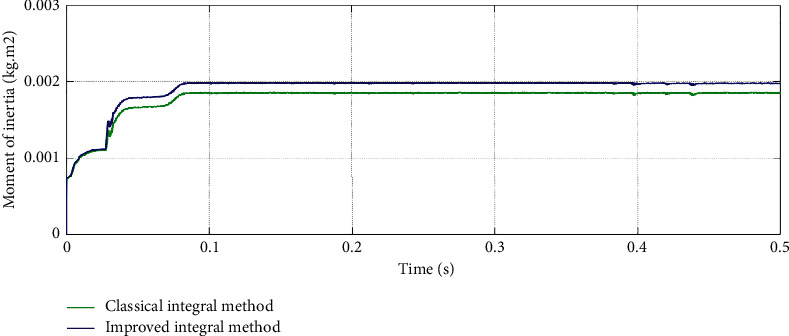
Comparison of inertia identification results based on classical and improved integral method.

Figure 10 shows the simulation experiment results of the classical fixed period integral method and the proposed improved sampling period update conditions integral method. The speed of the simulation experiment is also set at 1000 r/min constant speed. It can be seen that, compared with the classical integral method in which the average error is about 5%, the error of the improved integral method in which the average error is about 1% is smaller. The method has high accuracy and stability, which proves that it has good identification performance for inertia.

After real-time matching of parameters, the inertia is used to calculate the load torque combined with the improved SMO, adopting the saturation function deformation mode with boundary mutation. The speed is the simulated speed waveform mentioned above of the servo system position regulation mode start-stop motion. The load torque of the simulation experiment and the parameters of the SMO are the same as those set above.

**Figure 11:**
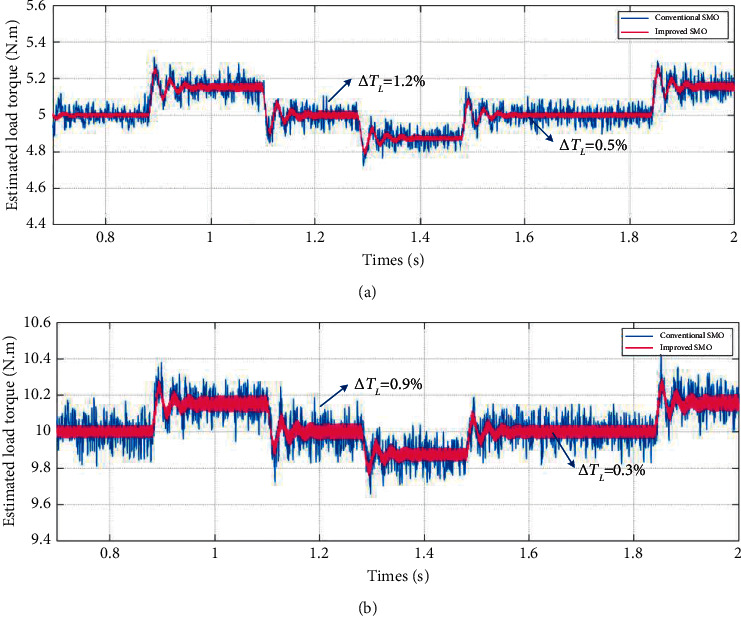
Partial experimental results of load torque estimation performance under the condition of variable boundary layer and variable speed combined with moment of inertia matching. (a) Load torque is 5 N·m. (b) Load torque is 10 N·m.

Figure 11 shows the conventional and improved SMO combined with parameter matching load torque estimation results under the given speed waveform. After the system runs smoothly, it is calculated from experimental data that the mean error of estimation for conventional method and proposed method is about 1.2% and 0.5%, respectively, when the load torque is 5 N·m as shown in Figure 11(a) and about 0.9% and 0.3%, respectively, when the load torque is 10 N·m as shown in Figure 11(b).

It is shown from the experimental results that after the inertia has been matched, the load torque varies with the actual speed and the waveform ripples are very small. Compared with the conventional SMO, the improved SMO has smaller estimation error and better dynamic response performance. It is proved that the improved SMO combined with moment of inertia matching method proposed in this paper can estimate the load torque effectively and accurately, and the chattering problem of the servo system is alleviated to a certain extent.

### 5.3. Tracking Performance and Stability of the Improved SMO

To further verify the superiority and ability of the proposed improved SMO combined with parameter matching, the tracking performance and antidisturbance performance of the observer are simulated.

**Figure 12:**
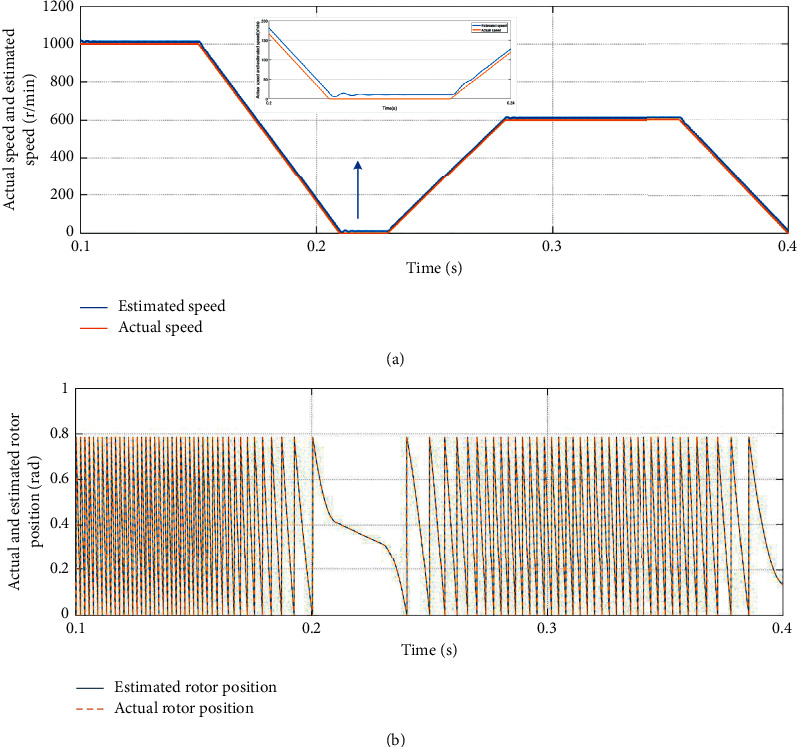
Tracking performance of the improved SMO combined with parameter matching. (a) Speed tracking performance. (b) Rotor position tracking performance.

Firstly, for the purpose of achieving high performance servo control system, accurate rotor position and speed information is generally required.  Figure 12 is the tracking performance of the improved SMO combined with parameter matching. The load torque of the simulation experiment is 5 N·m and the parameters of the SMO are as follows: *k*=1000, *l*=9, and *m*=10.  Figure 12(a) shows the actual speed and estimated speed of the simulate servo system position regulation mode start-stop motion speed waveform.  Figure 12(b) shows the actual and estimated rotor position simulation result.

It can be seen from the experimental results of tracking performance that the estimated speed and estimated rotor position can vary with the change of the actual speed and rotor position and the dynamic response performance is good. It is proved that the improved SMO combined with parameter matching in this paper has excellent tracking performance.

**Figure 13:**
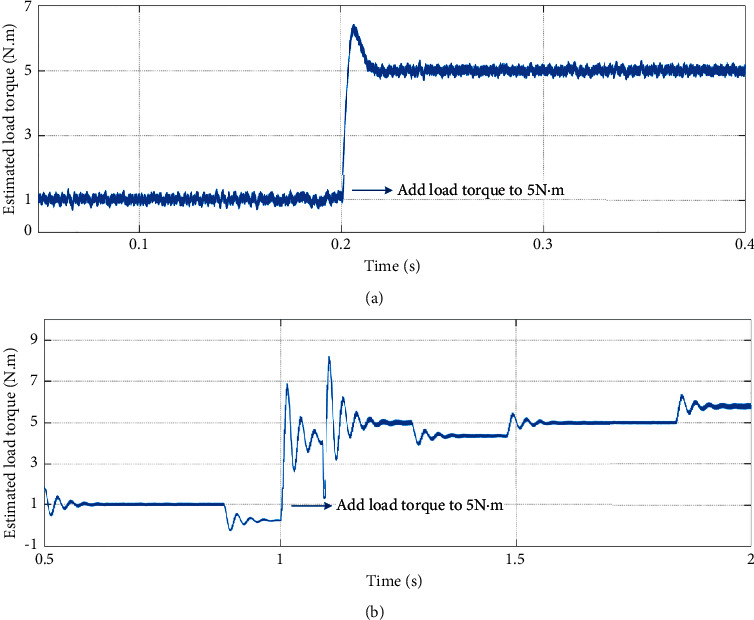
Stability of the improved SMO combined with parameter matching. (a) Constant speed. (b) Variable speed.

Secondly, the sudden load is added at constant and variable speed, respectively, to verify the antitorque disturbance ability of the improved SMO.  Figure 13 shows the stability test results of the improved SMO combined with parameter matching.  Figure 13(a) shows the constant speed of 1000 r/min with sudden load change experimental result that the initial load torque is 1 N·m and a sudden load torque of 5 N·m is added at 0.2 seconds.  Figure 13(b) shows the variable speed with sudden load change experimental result that the initial load torque is 1 N·m and a sudden load torque of 5 N·m is added at 1 seconds.

The simulation results show that, no matter at the condition of constant speed or variable speed, after a short fluctuation, the estimated load torque enables smooth tracking of actual load during sudden loads, and the errors and chattering are small. Therefore, it is proved that the improved SMO combined with parameter matching in this paper has excellent resistance to disturbance and can respond quickly when the load torque changes.

Through the above theoretical analysis and simulation results, the load torque identification method based on improved SMO combined with inertia matching proposed in this paper can effectively alleviate the chattering problem existing in conventional SMO and improve the identification accuracy of load torque. In the future research, we will focus on the torque fluctuation suppression, the parallel identification, and optimization of multiple mechanical parameters of the robot servo system.

## 6. Conclusion

In this paper, the problem of load torque identification for servo system is studied. Chattering phenomenon has been one of the problems affecting the performance of conventional SMO. Firstly, we propose an improved SMO that uses the boundary-variant saturation function deformation mode as the switching function to alleviate the chattering phenomenon in this paper. The boundary layer adaptively adjusts with motor speed changes and the RBFNN is used to adaptively tune the boundary layer gain. Meanwhile, the configuration problem of sliding mode gain and feedback gain is analyzed. Secondly, due to the fact that the moment of inertia is required for load torque calculation and the mismatch of it will cause calculate errors, a variable period integral method is used for inertia identification, and the inertia and load torque sampling calculation period are redefined to update each order. Finally, the corresponding experiments are simulated and compared to verify the correctness of the scheme. The experimental results show that the proposed improved SMO combines observer gain coefficient tuning and inertia matching can alleviate the chattering and reduce the estimation error adaptively during the identification process. Although the chattering phenomenon cannot be completely eliminated, the proposed SMO is still an effective, superior, and stable method.

## Figures and Tables

**Table 1:** Main parameters of the servo controller model.

Parameter	Value
Rated speed	1000 r/min
Rated power	600 W
Rated current	3.5 A
Rated torque	10 N m
DC link voltage	220 V
Pole pairs	4
Stator resistance	0.643 Ω
Stator inductance	8.5 mH
Flux linkage	0.175 Wb
Inertia of servo motor	2 × 10^−3^ kg m^2^
Friction coefficient of servo motor	0 N m s

## Data Availability

The data used to support the findings of this study are available from the corresponding author upon request.
